# Pseudo-Online BMI Based on EEG to Detect the Appearance of Sudden Obstacles during Walking

**DOI:** 10.3390/s19245444

**Published:** 2019-12-10

**Authors:** María Elvira, Eduardo Iáñez, Vicente Quiles, Mario Ortiz, José M. Azorín

**Affiliations:** Brain-Machine Interface Systems Lab, Miguel Hernández University of Elche, Avda. de la Universidad S/N, Ed. Innova, Elche, 03202 Alicante, Spain; maria.elvira01@goumh.umh.es (M.E.); vquiles@umh.es (V.Q.); mortiz@umh.es (M.O.); jm.azorin@umh.es (J.M.A.)

**Keywords:** Brain-Machine Interface (BMI), EEG, obstacle, gait

## Abstract

The aim of this paper is to describe new methods for detecting the appearance of unexpected obstacles during normal gait from EEG signals, improving the accuracy and reducing the false positive rate obtained in previous studies. This way, an exoskeleton for rehabilitation or assistance of people with motor limitations commanded by a Brain-Machine Interface (BMI) could be stopped in case that an obstacle suddenly appears during walking. The EEG data of nine healthy subjects were collected during their normal gait while an obstacle appearance was simulated by the projection of a laser line in a random pattern. Different approaches were considered for selecting the parameters of the BMI: subsets of electrodes, time windows and classifier probabilities, which were based on a linear discriminant analysis (LDA). The pseudo-online results of the BMI for detecting the appearance of obstacles, with an average percentage of 63.9% of accuracy and 2.6 false positives per minute, showed a significant improvement over previous studies.

## 1. Introduction

The rate of individuals affected by a motor disability is increasing due to the aging of the population and the growing number of chronic diseases, standing at 15% of the world population, according to the World Health Organization (WHO). It has been shown that rehabilitation treatment in the first six months after a stroke or a spinal cord injury is the most effective way to recover lost mobility, due to the plasticity of the nervous system during this time [1]. At this stage, however, traditional rehabilitation presents great difficulties for many patients because of their inability to perform the relevant movements or because of the excessive physical effort that therapy requires. For this reason, the use of exoskeletons during rehabilitation therapies presents great benefits. In addition, the use of Brain-Machine Interfaces (BMIs) allow a greater involvement of the patient during his/her rehabilitation and, consequently, a better recovery [2].

BMIs based on electroencelographic (EEG) signals record the brain activity in a non-invasive way and translate brain signals into commands for controlling external devices [3]. BMIs have been applied in rehabilitation for controlling upper-limb prosthetics [4,5]. In relation to the development of BMIs for commanding lower-limb exoskeletons, EEG signals have been analyzed in order to detect mental states related to walking [6].

On one hand, patients can use an exoskeleton in the chronic phase to be assisted in movement [7,8]. This allows them to increase their independence and mobility. On the other hand, the acute phase of rehabilitation is when a better improvement of the functional capabilities of a patient can be acquired. Therefore, the combination of an exoskeleton and BMI could also be useful during therapy, due to the cognitive involvement of the users, with a more intuitive, clear and dynamic control.

EEG signals can be used for obtaining relevant information about patient intentions or specific motor mental processes. In this regard, several works analyze the detection of starting or stopping gait from EEG signals based on voluntary intention of the user [9,10,11]. Continuous gait motor imagination has also been used as a paradigm to control the gait of an exoskeleton [12].

Moreover, there are other mental processes of interest which could be useful in controlling an exoskeleton, such as the detection of speed changes or direction changes [13]. All the former research has been used to create commands to control an exoskeleton. However, the reliability of the BMIs must increase before extended real-time applications, and especially the false positive rates must be reduced.

Another important aspect when commanding an exoskeleton is to keep high safety conditions. In the case that an unexpected obstacle appears, it is necessary to stop the device as soon as possible. Exoskeletons used to have an emergency button for this purpose, but it requires an active action by the subject. This action is not instinctive and it could be affected by the medical condition of the subject and by response time. An alternative option could be the integration of an automatic image processing system to detect obstacles and generate the stop command [14]. However, this option requires additional hardware and it is more computationally extensive. Moreover, it requires not only the detection of the obstacles, but also the interpretation of whether or not it is an obstacle that really requires a stop. For example, Bi et al. use a vision system to detect obstacles during driving situations [15]. In this work, the visual system is also combined with obstacle detection through EEG signals.

The use of cerebral information is based on the detection of different potential patterns when an obstacle appears and the person voluntarily decides to react by stopping or not. This analysis could allow a transparent detection by the subject, performing the detection faster than the user’s physical reaction. Some studies have already analyzed brain signals through error-related potentials (ERPs), studying visually in the frequency domain the response to an obstacle between aggressive and gentle drivers when using a driving simulator [16]. If the detection is performed soon enough after perceiving the obstacle, an emergency stop can be performed, improving safety. This includes not only the visualization of the obstacle, but also the decision to react to it. Therefore, it is the person who will determine the type of reaction to the obstacle, avoiding the need of interpretation by the system that visual paradigms require.

The detection of the intention of a subject to stop after the appearance of an unexpected obstacle has been analyzed in previous works [17,18]. In these previous studies, brain signals were analyzed when an unexpected external disturbance appeared while walking on a treadmill. The disturbance was simulated by a laser line and the user had to suddenly stop their gait in response to the obstacle. It was found that when averaging several events, a potential appeared in the electrodes of the fronto-central area before the real stop of the user, allowing anticipation of the actual stop. However, those events must be detected in a single trial in order to generate commands in real time. The offline analysis of the features of the potential through common spatial patterns (CSP), slope or polynomial fit allowed the classification by linear discriminant analysis (LDA) with a maximum accuracy of 80%. As a last step, a pseudo-online analysis with a sliding window of a half second to detect the events was performed. Even if the initial results were promising, the false positive rate still remained very high (6.7) and accuracy dropped to 30% [17]. A low false positive rate would allow someone to send a stop command to an exoskeleton in the case of an emergency event, making possible its implementation in real time in a reliable, useful and safe way [19].

The goal of this work is to significantly improve the results of previous work. Inertial measurement units (IMUs) are used to measure the real moment of a stop in order to compare it with the detection of a stop intention by the system. Moreover, new methods have been explored for personalizing the electrode selection and to determine the data window with more relevant information for the model creation. New features allow a better differentiation between the potential after and before the event. Results are tested by a pseudo-online analysis which provides a more similar approach to future real-time implementation with an exoskeleton. The paper shows the pseudo-online results obtained by nine healthy subjects.

## 2. Materials and Methods

The following section describes the software and hardware that have been used in the experiments, as well as the experimental procedure. This includes: the methods used for pre-processing the EEG signals, extraction of features, and their classification. Furthermore, the methods used for an accurate monitoring of the movement of the person through inertial measurement units (IMUs) are also detailed. Finally, the strategy used to get the pseudo-online results is also defined.

### 2.1. Experimental Set-Up

Two commercial amplifiers (g.USPamp from the g.Tec company, Graz, Austria) have been used for registering the EEG signals. Each amplifier registers 16 input channels, so a total of 32 electrodes are registered at a sampling frequency rate of 1200 Hz. The amplifiers include a notch hardware filter at 50 Hz. Both devices have been synchronized using the g.INTERsync module. The electrode distribution follows the International System 10/10 [20], and they are distributed over the scalp following this order: Fz, FC5, FC1, FCz, FC2, FC6, C3, Cz, C4, CP5, CP1, CP2, CP6, P3, Pz, P4, PO7, PO3, PO4, PO8, FC3, FC4, C5, C1, C2, C6, CP3, CPZ, CP4, P1, P2 and POz. The reference has been positioned on the right ear lobe, and AFz is used as ground.

Furthermore, the Tech MCS V3 equipment from Technaid (Arganda del Rey, Spain) has been used for monitoring the movement of the subject. The IMUs are used to detect the moment when the subject actually stops. This allows researchers to mark the time of the windows of analysis for the moments prior to the obstacle appearance and when the subject reacts to the stimulus and stops. The Tech MCS V3 device uses 7 IMUs, registering up to 19 parameters such us acceleration or gyros at a frequency sample of 30 Hz. They are distributed in the body as follows: one IMU is placed on the lumbar area, and three IMUs are placed on each leg on the right/left thigh, right/left shin and right/left foot.

Both EEG signals and motion information are registered and synchronized using a Matlab API. Matlab has also been used for data processing and analysis.

**Figure 1:**
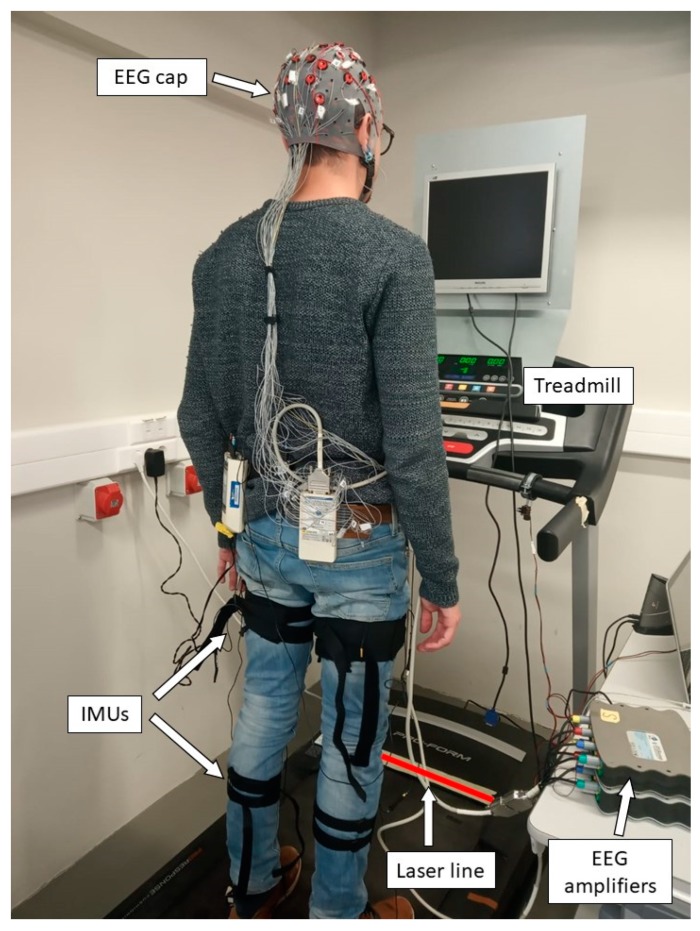
Experimental set-up. The subject stands on the treadmill wearing the inertial measurement units (IMUs) for registering movement and the electroencelographic (EEG) cap for registering EEG signals. The subject keeps a normal gait of 2 km/h for 2 min, while a laser line is projected randomly onto the front of the treadmill.

Additionally, a treadmill Pro-form Performance 750 has been used to keep a constant and controlled velocity during the trials. A laser line projected onto the front of the treadmill, with a wavelength of 635 nm (red color) and an output power of 3 mW, has been used to simulate the appearance of sudden obstacles. The laser line is activated by a trigger out of the g.USBamp device, which also allows labelling of the EEG events. Figure 1 shows the experimental set-up.

### 2.2. Experimental Procedure

The experimental procedure is as follows:

1. First, the full procedure is explained to the subjects and all the equipment is set up. Subjects see how the obstacle appearance works through laser activation. Before the experiment starts, subjects get used to stopping when they see the laser line appear. As the treadmill is moving slowly, it is safe to stop for a second and a half and to resume their gait without falling from the platform. Nevertheless, a safety belt is set on the subject to stop the treadmill automatically if the subject moves a certain distance backwards. In addition, a researcher in charge of the experiment is placed behind the treadmill during the experiment to avoid any possible unsafe situation.
2. Then, subjects perform 10 trials to complete a full session. For each trial, the next steps are followed:
  - First, the subject stands on the treadmill without any movement, while the software is connected to the g.Tec equipment and the IMUs are calibrated.
  - Next, the subject starts walking on the treadmill at a constant velocity of 2 km/h and 0 degrees of inclination. Once the person is walking safely and in a stable manner, the data acquisition starts.
  - Each trial lasts 2 min. During this time, the laser line is projected randomly during one second. The interval between two successive stimuli varies between six and nine seconds. Therefore, the total number of lasers that appear on each trial is between 12 and 14. The subject is instructed to suddenly stop when the laser is visualized and then resume gait after a couple of seconds as previously explained.

### 2.3. Stop Detection Method Through IMUs

As mentioned before, 7 IMUs have been distributed on the body. The module of the acceleration of the axes X, Y and Z (3 of the 19 outputs of the IMUs) is calculated. After that, the continuous wavelet transform (CWT) [21], with a scale range of 1:64 is applied. Then, a parameter is calculated as the sum of the CWT coefficients in the range 3:30 (where the frequency components of interest are).

In order to make a decision, an initial threshold is needed. This is determined as the mean of the parameter of the first trial of each subject. Each trial is analyzed with a sliding window of 20 samples (0.67 s) shifted at a 3 samples pace (0.1 s). If the parameter computed for each window is less than half of the threshold, then it is considered that there is a significant decrease in the signal, and a stop is computed. As the windows analysis overlaps, a detection is only considered if there is a significant decrease in the current epoch and not in the previous one. Finally, the threshold is updated for each window, averaging it with the signal value of the previous window.

### 2.4. Offline Model Creation

**Figure 2:**
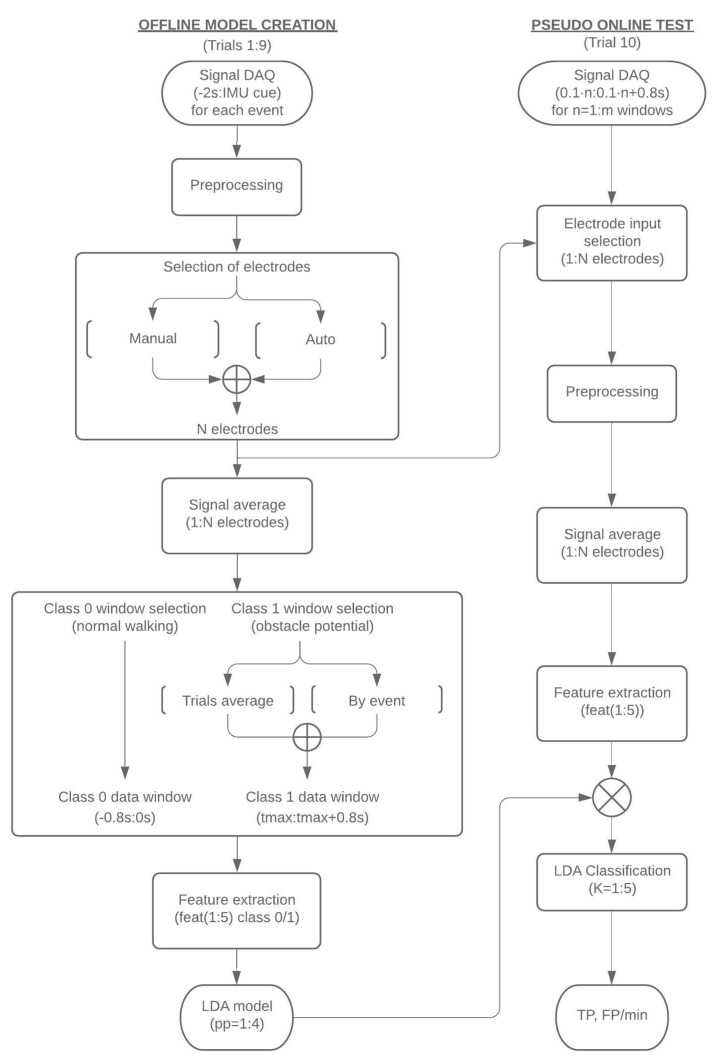
General scheme of the EEG signal acquisition applied to each window. Left shows the procedure for the offline model creation. Right shows the pseudo-online test procedure.

This subsection shows the different steps and alternatives considered to create the model for classification of the EEG signals: preprocessing, electrode selection, time window selection and classification. Figure 2 (left) shows a general diagram of the procedure applied.

First, in order to create a suitable model that allows the detection of obstacles, it is important to determine a proper window of analysis. This window must cover the time from when the subject is walking normally to when an actual stop is detected by the IMUs. Then, the electrode information corresponding to this period of time is preprocessed and subsequently the selection of a significant group of electrodes is averaged for them. Two different classes are considered for the model creation, depending on the performed action: normal walking (class 0) and response to the obstacle (class 1). For each event of an obstacle appearance, features are extracted from the data of both classes for model creation. The trials considered for model creation correspond to the first 9 trials of a session (Figure 2, left), leaving the 10th trial for testing the model in a pseudo-online scenario (Figure 2, right). In the case of the pseudo-online analysis, the preprocessing is only applied to the personalized selection of electrodes of each subject obtained during the model creation.

#### 2.4.1. Preprocessing

In the case of the model creation, for each event, the period of analysis starts 800 ms before the laser activation and ends before the IMU’s detection of the stop. To increase the noise-to-signal ratio some preprocessing must be applied to the signals.

Based on a previous work where motion artifacts were analyzed during walking conditions, it was concluded that the noise is mainly focused on peripheral areas corresponding to the scalp locations more sensitive to conductivity changes [22]. Moreover, in this previous work, periphery electrodes were discarded after analyzing all the electrodes and verifying that they were more affected by artifacts due to cap features, e.g., poor contact with the scalp when walking [17].

This is why, from the initial 32 electrodes, those located on the periphery of the head, and therefore more possibly affected by movement artifacts, have been discarded: FC5, FC6, CP5, CP6, PO3, PO4, PO7, PO8, C5 and C6.

Then, to the remaining 22 electrodes, a band-pass filter from 0.4 to 3 Hz has been applied as the studied potentials are in a low-frequency range. Finally, in order to remove possible artifacts, those signals with standard deviation greater than 40 µV have been removed. This is an important aspect to assure that the model creation uses only suitable EEG information.

#### 2.4.2. Selection of Electrodes

There are four main lobes in the brain: frontal, temporal, parietal and occipital [23]. The EEG pattern to be looked for corresponds with the response to an unexpected visual stimulus, which is also associated with the intention of stopping. Therefore, the brain areas of interest for analysis are the motor, sensory and occipital area, which are located respectively in the frontal, parietal and occipital lobes. After removing the peripheral electrodes indicated in Section 2.4.1, the remaining 22 electrodes are part of the aforementioned areas. This way, the electrodes registered are: Fz, FC1, FCz, FC2, C3, Cz, C4, CP1, CP2, P3, Pz, P4, FC3, FC4, C1, C2, CP3, CPz, CP4, P1, P2, POz.

However, not all the electrodes are used for the classifier and a subset of the electrodes is selected based on the magnitude of the variation of the potential after a laser activation (Figure 2, left). This selection has been carried out in two different ways: manual and automatic.

**Figure 3:**
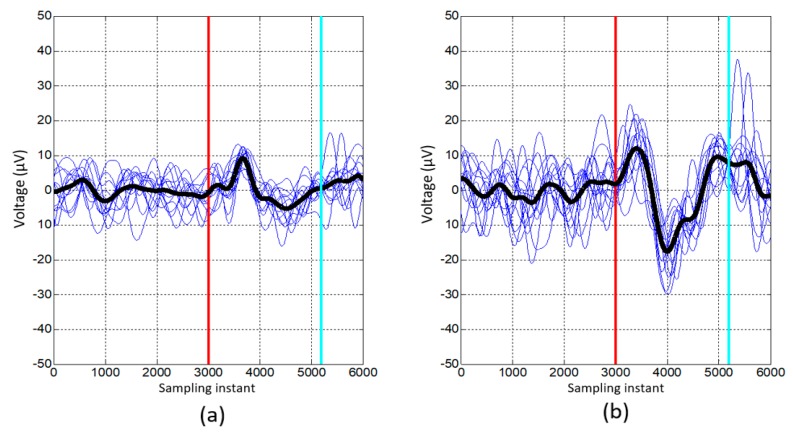
Comparison between the signal obtained by choosing all the electrodes of the mentioned areas (**a**), and the one obtained including only the signal of the electrodes chosen individually (**b**). On the X axis, the sampling time for a frequency of 1200 Hz appears. On the Y axis the value of the voltage in µV is represented. The laser appears at the sampling instant 3000 (at 2.5 s) and is represented by the red vertical line, picking up the signal for the 2.5 s before the laser and the 2.5 s after. In addition, the vertical line in cyan blue represents the moment in which the real stop of the person occurs (averaging all the stops made in the session represented and detected by IMUs).

The manual selection method consists of visual observation of the signals after the laser appearance for each electrode. The selected electrodes are the ones that from inspection show a higher distinctive potential. This procedure takes usually less than five minutes. Figure 3 represents an example of the inspected signals for one of the subjects and shows the comparison between the averaged signal (black line) obtained by choosing the 22 electrodes (a), and the one obtained by manual selection (b). It can be seen that after manual selection, the averaged signal is more homogeneous and there is a more significant change after the laser line appearance (red line) and before the real physical stop of the subject determined by the IMUs (cyan line).

The automatic selection of the electrodes tries to reproduce the previous criteria. The objective is to eliminate those electrodes that contribute less to the expected potential. The procedure of this automatic selection method can be defined as follows:

1. The procedure starts with the preprocessed signals from −2 s to the IMU’s cue for each event and the 22 electrodes (N = 22).
2. N different combinations of electrodes, discarding one electrode for each combination, are analyzed.
3. For each combination, all N–1 electrodes are averaged for each individual event each time the laser line appears (similarly to the black line in Figure 3).
4. As the pattern expected is a first positive deflection followed by a negative deflection, the method locates the first maximum voltage ($max_{láser}$) after the laser appearance, and locates the minimum voltage ($min_{láser}$) in the first second after this maximum. In addition, the maximum voltage ($max_{walk}$) and the minimum voltage ($min_{walk}$) of the signal during the two seconds of normal walking before the laser appearance are also calculated. These values are assessed for each of the laser activations. After that, the relation between the amplitude of the signal after and before the laser is obtained as:
  (1) $$dif=\frac{max_{láser}-min_{láser}}{max_{walk}-min_{walk}}*100$$
5. Then, a single value for each of the combinations of the electrodes is calculated as:
  (2) $$dif_{total}=average\left(dif\right)-standard\;deviation\;\left(dif\right)$$
6. Next, the maximum *dif_total_* value (corresponding to a certain configuration of electrodes) is analyzed. If this maximum value is higher than the maximum value from the previous iteration (for the first iteration it would be compared to the original 22 combination), the electrode not participating in the combination is discarded and N = N–1.
7. This process (steps 2 to 6) is repeated for successive N–1 combinations of electrodes, i.e., while the first iteration tests combinations of 21 electrodes, the second tests for combinations of 20 electrodes, third of 19 and so on.
8. Iteration stops when the maximum *dif_total_* of the iteration has a lower value than the one from the previous iteration.
9. In order to be sure that the removed electrodes are not relevant, a second revision is performed.
10. This second revision is done through trying a subset of electrodes, discarding one by one the discarded electrodes. Steps 2–5 are repeated keeping the electrode only if its *dif_total_* is higher than the one obtained by Step 8, or definitively discarding it if its lower.

#### 2.4.3. Personalized Selection of Time Windows

According to the conclusions obtained in [17], it is known that after a visual stimulus is followed by a reaction, a positive deflection in the EEG signal 700 ms before the reaction of the person, followed by a negative deflection 400 ms before that reaction, can be observed. However, high variability between people’s reaction times should be considered. Therefore, in order to select a suitable time window for modeling the EEG potentials and for improving the results, the time window is selected for each subject individually (Figure 2, left). That is when the greatest variation of the amplitude of the EEG signal is observed. This will improve model creation.

**Figure 4:**
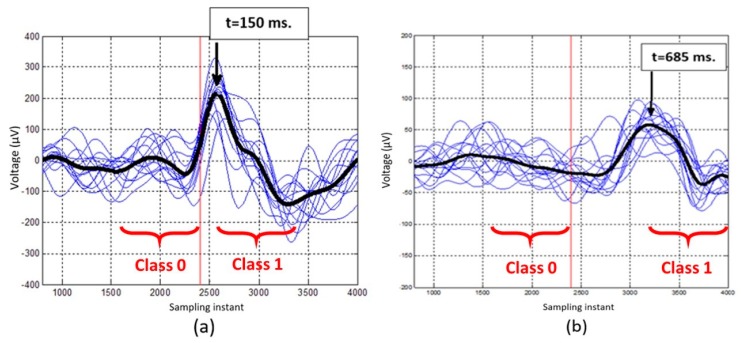
Temporary lag between the positive deflection in subjects S8 (**a**) and S6 (**b**). The sampling time (*f* = 1200 Hz) is represented on the X axis. The amplitude of the signal obtained by adding the potential in the electrodes chosen for each subject individually, in µV, is represented on the Y axis. The blue lines represent this sum signal in different laser activations of the same session and in black are the average of all of them. The vertical red line represents the appearance of the laser.

Figure 4a,b show, as an example, the temporary lag between two subjects, S8 and S6. The electrodes selected in the previous step are averaged and each laser activation is shown together with the average. While in the first case the maximum of the average signal is produced 150 ms after the laser, for the second case the maximum is located at 685 ms. Considering this situation, two possible solutions are proposed for selecting the beginning of the class 1 window.

The first alternative consists of calculating on average the moment in which the greatest variation in the amplitude of the EEG signal occurs. This is calculated by obtaining the average of all events (as shown in Figure 4), and detecting the moment of the maximum of this average signal.

The second alternative tries to create a personalized model, locating the maximum of the signal in each event in the range of 2 s after the laser appearance.

Therefore, the time window that represents the class 1 (laser) for obtaining the model will be chosen starting in the time when the maximum is detected and will have a length of 800 ms. In contrast, the time window of class 0 (non-laser) will be chosen equally for all subjects lasting also 800 ms just before the laser appearance. This way, the area of greatest difference between class 1 (laser) and class 0 (non-laser) can be captured.

#### 2.4.4. Feature Extraction

After the selection of the subset of electrodes and the 800 ms time windows for class 0 and 1, feature extraction is performed (Figure 2, left). In all cases, the features have been obtained from the average of the signal of the electrodes chosen for each subject, which is called *f*1.

From this signal *f*1, a vector with 5 features has been extracted:

1. Area included under the curve of the absolute value of *f*1 derivative:
  (3) $$feat\left(1\right)=\int abs\left(f1^{\prime }\right)$$
2. Variance of *f*1 derivative:
  (4) $$feat\left(2\right)=var\left(f1^{\prime }\right)$$
3. Amplitude of *f*1 derivative:
  (5) $$feat\left(3\right)=max\left(f1^{\prime }\right)-min\left(f1^{\prime }\right)$$
4. Amplitude of the accumulative sum of the absolute value of *f*1 along a window:
  (6) feat(4)=max(sum(f1))−min(sum(f1))
5. Maximum cross correlation between *g*1 and *f*1 in each case (*g*1 is the mean of all the *f*1 obtained in each particular laser appearance):
  (7) $$feat\left(5\right)=max{\left(f1⋆g1\right)}_{i}$$

The features vector is obtained for all window classes 0 and 1, and is properly labelled.

#### 2.4.5. Classifier

For the distinction between the 2 classes, a linear discriminant analysis (LDA) has been chosen as the classifier, which transforms a set of α-dimensional data into a δ-dimensional subspace (with δ < α), maximizing the distance between the means of each class and, simultaneously, minimizing their dispersions [24,25] (Figure 2e).

The discrimination function is as follows:

(8) $$h_{\beta }\left(x\right)=\{\begin{array}{c} \left(\beta^{T}\cdot x+\beta_{o}\right)\geq 0\to 1\\ \left(\beta^{T}\cdot x+\beta_{o}\right)<0\to 2 \end{array}$$

(9) $$\beta =\sum^{-1}\left(\mu_{1}-\mu_{2}\right)$$

(10) $$\beta_{o}=-\beta^{T}\cdot \left(\frac{\mu_{1}+\mu_{2}}{2}\right)+ln(\frac{\pi_{1}}{\pi_{2}})$$

where *β* is the vector of classification parameters, *β_o_* is the bias term, ∑ is the clustered covariance matrix, *μ*_1_ and *μ*_2_ are the average vectors of class 1 and class 2, and *π*_1_ and *π*_2_ are their prior probabilities [26]. As these probabilities are not known a priori, they are also analyzed in the results section for a prior probability (*π*) assigned to class 0 of *π* = 1:4 versus *π* = 1 to class 1.

### 2.5. Pseudo-Online Analysis

In order to achieve a more realistic evaluation of the BMI, a pseudo-online classification is performed using the last trial of each session.

For this purpose, a sliding window from EEG signals of 0.8 s has been analyzed, shifting each one by 0.1 s. Each window was analyzed following the procedure in Figure 2 (right) and is classified as to whether it belongs to an event of walking (class 1) or response to the obstacle (class 0).

The process is applied only to the electrodes selected during the model creation. Since the band-pass filter can distort the sides of short time windows, the length of the window is extended to the previous seconds of each moving window. Then, after applying the band-pass filter, only the last 0.8 s are kept for the next steps. Then, the data of the selected electrodes are averaged and their features are extracted. Finally, the LDA model is applied to obtain the classification of each moving window as class 0 or 1.

Due to the overlapping of windows (0.7 s), there is redundant information that can cause the detection of several successive windows as class 1, when for instance only one has been produced [27]. Therefore, a K value that represents the number of positive detections (class 1), must be taken into account to assure a correct one. This parameter has been tested for values of K from 1 to 5. For example, if an isolated detection is performed with K = 2, it will not be considered, as it would require at least 2 consecutive detections to its computation. A correct selection of K helps to reduce the number of false positives.

In addition, the real-time algorithm for detecting the stop through the Tech MCS equipment described in Section 2.3 has been included in the pseudo-online test. Due to the structure of the test, when the person restarts the gait after a stop, a noise is generated on the EEG signal. This distortion affects the BMI system, as it could be detected as a false positive. However, as the obstacle detection must be only considered when the subject is walking, the predicted class is automatically set to 0 during the windows associated with the 1.5 s after the IMU’s detection of a stop. This period of time is not considered for the total count of seconds, in order to not affect the FP/min rate.

### 2.6. Subjects

Nine healthy subjects, with ages between 21 and 54 years, without any neurological disorders and with normal vision have participated in the experiments. The experiments were approved by the Ethics Committee of the Miguel Hernández University of Elche (Spain). All subjects were informed and signed informed consent according to the Helsinki declaration.

## 3. Results

This section starts describing the results of the stop detection through the IMUs. This is a critical result, as the feedback of the IMUs provides the tool used for the definition of a real physical stop. Section 3.2 shows the subset of electrodes obtained by manual selection as explained in Section 2.4.2. From Section 3.3 onwards, the results cover the different analysis considered for the detection of the stop intention through EEG. Results are obtained from the pseudo-online analysis of the last trial of each session. The parameters computed include the true positive rate (TP) and the false positive rate (FP/min). The different subsections correspond to some of the alternative paths shown in Figure 2. The first default analysis (Section 3.2) uses a manual selection of electrodes, the average of trials for the window selection of class 1, a prior probability of 4 and K values from 1 to 5. From those default results, successive analyses study alternative paths such as changing the prior probability of the classifier, selecting the class 1 windows by event or using an automatic selection of the electrodes. Finally, the time of detection by the algorithm is compared to the time when the stop is computed by the IMUs, in order to assure that the detection is assessed before the real stop happens.

### 3.1. Results of the Stop Detection through the IMUs

Table 1 shows, for each subject, the percentage of stops correctly detected through IMUs (TP: true positive) and the percentage of stops not detected (FN: false negative). Both values have been calculated on average for all the sessions of each subject. The number of false positives is also included, considering FP as the cases where a stop is detected but there is not a real one. Furthermore, the average time for each subject to detect the stop from the laser activation is shown.

From the results, it can be asserted that the algorithm properly detects the stops, with a TP percentage of 92.5% ± 7.0% and only 0.1 ± 0.2 FP. There are only false detections for 2 of the subjects (S5 and S6) while the rest of them have 0 false detections.

Regarding the reaction time for each subject, the values are in the range of 1.6 to 2.1, which could be considered a high variability range. In this sense it must be considered that all the subjects do not stop in the same way. Some of them stop their gait immediately after seeing the laser, while others go one step further before stopping. This could explain this variability, in addition to the different reaction times of each person.

It is important to remark that the objective of the research is to detect the intention of the subject to stop when an obstacle appears. Therefore, the time showed in Table 1 must be lower than the needed time by the developed algorithm to detect a stop intention.

### 3.2. Manual Electrode Selection

Table 2 shows the list of electrodes selected for each subject with the manual method. Furthermore, the maximum of the average signal is included in the third column, in milliseconds, determined by the addition of the selected electrodes in all laser appearances.

From the results some conclusions can be obtained. First, in 8 out of 9 subjects there is an activation of the electrode FCz, which reflects that an error-related potential is being generated when the obstacle appears [28]. Also, electrodes Cz and Fz, located in the same area (fronto-central area) appear in most of the subjects. Additionally, there is an activation of the motor area, with the inclusion of electrodes such as FCz, FC2, C1, C2, C3 and C4 due to the intention of a stop. Finally, the occipital electrodes such as P1, P2, P3, P4, POz and Pz are also activated due to the visualized obstacle. Both behaviors could be expected since we have a visual stimulus (laser), to which the subject reacts, sending an order to stop.

### 3.3. Results of the Pseudo-Online Analysis with Initial Configuration

Table 3 shows the results obtained in the pseudo-online analysis of the tenth trial. The default configuration considered is based on the manual electrode selection and the time windows selection for class 1 after the maximum time showed in Table 2. The prior probability assigned to class 0 (non-laser) is 4 versus 1 for class 1 (laser).

A detection is considered successful when it happens in the interval between the appearance of the obstacle and the average reaction of the subject. The pseudo-online analysis is calculated for K values between 1 and 5. Results for K = 1 are not detailed in the tables since the FP/min are too excessive to be considered as acceptable.

The criteria which has been followed for considering the best result is to keep a FP/min rate lower than 4 with the highest TP value. Following this rule, Table 3 shows in bold text the best result for each case and subject. Results show a mean TP value of 59.5% ± 25.9% and an average value FP/min rate of 2.7 ± 1.3 The K values depend on the subjects with values from 2 to 4.

### 3.4. Results Varying the Prior Probability of the Classifier

Table 4 shows the comparison between the TP and FP/min rates for different values of prior probability π = 2:4 assigned to class 0 (non-laser) versus π = 1 to class 1. To condense the information, only the results of the optimum K value are shown for each subject.

Results for equal prior probabilities (1 vs. 1) for both classes have not been included because of the high FP/min obtained by all the subjects.

According to these results, prior probability of 3 for the class 0 offers the best relation between TP and FP/min rate, so the next analysis will use this probability to calculate the results.

### 3.5. Results when Modifications are Made to the Best Case

The results in this subsection take into account two alternatives regarding time window selection and automatic electrode selection.

#### 3.5.1. Results for Time Windows of Class 1 Obtained from a Particularized Maximum by Event

Table 5 shows the results obtained for capturing the time windows for class 1 locating the maximum of the signal for each time window, according to the explanation given in Section 2.4.3. Results are generally worse with this alternative, except for the subject S1, who obtains a significant improvement. For the rest of the subjects TP has a value of 46.6% ± 27.8% and FP/min of 2.7 ± 0.8. This reflects a decrease of nearly a 15% in the obstacle detection, while the FP/min rate only decreases by 0.1. Therefore, it is not worthwhile to particularize the class 1 window by event.

#### 3.5.2. Results Obtained with the Automatic Selection Electrodes

Table 6 shows the list of the electrodes obtained with the automatic selection as it was explained in Section 2.4.2, as well as the time instance from which the time window for class 1 is captured for creating the model, according to the mean obtained from all the laser appearances.

If this selection is compared with the one obtained manually, it can be seen that with the automatic method, in 8 out of 9 cases the selection includes a lower or equal number of electrodes. Additionally, as it happens with the manual selection, the electrodes that appear more frequently are again the ones related to the error-related potential: FCz, Fz (which appear in all the subjects), Cz, FC1 and FC2. The electrodes located in the occipital zone (P1, P2, P3, P4, POz and Pz), where visual information is generated, also seem to contribute to the generation of a distinctive potential in most of the cases. Electrodes located in the motor area (C1, C2, C3 and C4), appear only in subject S7, so the potential generated for the stop intention is weaker, contributing less to the detection of the potential in the automatic selection method.

Table 7 shows the results obtained with the pseudo-online analysis. Only the results for the best value of K are shown. The right side of the table shows the best results obtained with the manual selection method for comparison.

It is difficult to consider which electrode selection performs better. Two out of the ten subjects, S1 and S8, improve with the automatic selection. However, in the case of S4 and S9, the results get considerably worse. Subject S3 obtains exactly the same rates, while in the rest of the cases it is not clear as to whether the results improve or worsen, because a TP rate increase is correlated to a higher FP/min and vice versa.

As a criterion for its application in real time, the electrode selection could be first done using the automatic selection, as it is an unsupervised, faster method. After the model and selection is created, a new trial could be done to see if the TP is higher than 60% and the FP/min is lower than 3.0. If they are, the automatic selection is used, and if not a supervised manual selection is done and tested in pseudo-online analysis to see if the results could be improved.

### 3.6. Analysis of Instant Time Detection Versus Obstacle Appearance and Physical Stop Detection

In this subsection the instance after the laser appearance, when the BMI detects the intention to make a stop through EEG signals, is analyzed. This instant is compared with the instant when the IMUs detect the stop. With the difference between these two moments it can be stated whether the stop intention is detected before the real stop of the person. For instance, this could be helpful in the command of an exoskeleton, allowing the subject to stop it faster than the reaction time.

Table 8 shows those results taking into account the optimal value of K obtained for each subject with a prior probability π = 3 for the class 0 and with the manual selection of electrodes. The time interval between the detection of a stop through the IMUs and the classification of class 1 through the BMI (2nd column) are shown. Table 8 also shows the interval between the obstacle appearance and the obstacle detection through the BMI (3rd column). Both intervals are calculated averaging all the time differences obtained with the different laser appearances. In all the cases, the detection of the potential has been produced before the real detection by the person. This means that the BMI has been able to predict the detection of the subject.

The average anticipation is 0.9 ± 0.2 s, which could be considered enough anticipation to send a command to an exoskeleton and stop it in the case of an obstacle appearance.

Regarding the time interval between the laser appearance and the detection through the EEG signals, an averaged value of 1.2 ± 0.2 s is obtained. In this case, however, a great variability between subjects is shown. In fact, it must be appreciated that there is a relation between the instant when the maximum of the EEG signal was observed in each subject and the mean value when the intention to stop is being detected through the pseudo-online analysis. For instance, in S4 the peak of the signal appears 720 ms after the laser appearance (the highest value) and has the highest time interval (1.5 s).

## 4. Conclusions

This paper has evaluated new methodologies for detecting the appearance of unexpected obstacles while walking using EEG signals. Since the final goal is to propose a method that can be used in real-time tests, the chosen alternative cannot involve excessive time for training the BMI system and obtaining the model. That is why it has been decided to personalize some of the algorithm decisions, due to the improvement of the results, and generalize others to reduce the time needed to create the model. The results have been tested in a pseudo-online scenario which is a more realistic analysis and is suitable for its future application in combination with an exoskeleton in real time.

Regarding the selection of the electrodes it is not possible to conclude which method, manual or automatic, achieves better results. Therefore, the criterion consists of applying the automatic faster selection in an initial stage, and depending on the results obtained, applying a second manual selection for trying to improve the results. The selection of the class 1 data for the model creation performs better when it is based on the maximum of the average signal of all events per subject instead of personalizing it by event. The results of applying different prior probabilities for the LDA classifier indicates that π = 3 is the most beneficial one in general terms for the class 0. Finally, the K value for consecutive detections should be personalized during the model creation between values of 2 and 5 for each subject, since the results vary widely depending on the subject.

Following all these considerations, the best results obtained for each subject according to all the tested variations are shown in right part of Table 9. The second column (M/A) indicates if a manual or automatic solution for electrode selection has been followed. This allows us to compare the results with previous work [17], showed in the left part of Table 9, where a polynomial feature extraction was performed. A significant improvement in the percentage of true positives can be observed, from 30.0% to 63.9%, and also a clear reduction in the FP/min (from 6.7 to 2.6). In addition, it is possible to affirm that the BMI system is capable of detecting the intention to stop of the person in a time prior to the actual stop, sufficient enough to be able to send an order to the exoskeleton in a reasonable time.

Future works will focus on performing real-time experiments by applying the procedure in order to move to the final stage by testing this architecture with an exoskeleton. It will also be analyzed if a lower number of trials to create the model allows us to obtain similar or better results in order to decrease the needed training time. However, there is still work to do in order to increase success percentage and to reduce as much as possible the FP/min. This is the most critical parameter for its implementation with an exoskeleton.

## Figures and Tables

**Table 1:** Results of stop detection through IMUs.

Subject	TP (%)	FP (%)	Average Time (s)
**S1**	92.3 ± 5.1	0.0	1.9
**S2**	82.7 ± 9.9	0.0	2.0
**S3**	99.2 ± 2.4	0.0	1.8
**S4**	88.1 ± 11.5	0.0	2.1
**S5**	95.9 ± 8.1	0.6	1.9
**S6**	82.3 ± 10.0	0.1	2.1
**S7**	96.9 ± 4.0	0.0	1.6
**S8**	96.2 ± 9.8	0.0	1.6
**S9**	99.2 ± 2.6	0.0	1.9
**Mean ± σ**	92.5 ± 7.0	0.1 ± 0.2	1.9 ± 0.2

**Table 2:** Manual electrode selection and maximum location for each subject. Electrodes coincidental with automatic electrode selection in bold text.

Subject	Manual Electrode Selection	Maximum Location (ms)
S1	**Fz**, FC1, **FCz**, **FC2**, **Cz**, **P1**	465.8
S2	**Fz**, FC1, FCz, **FC2**, CP1, **Pz**, **P4**	329.3
S3	**Fz**, **FCz**, **Cz**, POz	414.2
S4	**Fz**, FC1, FCz, FC2, C3, Cz, CP1, CP2, P3, Pz, FC3, C1, C2, CP3, CPz	719.2
S5	**Fz**, FC1, FCz, **FC2**, Cz, C4, **P1**	547.5
S6	C4, CP2, Pz, **P4**	665.8
S7	**Fz**, **FC1**, FCz, Cz, CP2	501.7
S8	**Fz**, **FC1**, FCz, FC2, C3, Cz, C4, CP1, CP2, P3	132.5
S9	**Fz**, FC1, FCz, FC2, **P1**, P2	523.3

**Table 3:** Results for the pseudo-online analysis with different values of K. Best values according the criteria (bold).

	K = 2		K = 3		K = 4		K = 5	
Subject	TP	FP/min	TP	FP/min	TP	FP/min	TP	FP/min
**S1**	69.2	8.8	61.5	4.4	**69.2**	**2.9**	38.5	1.5
**S2**	46.2	4.9	**30.8**	**1.4**	7.7	0.0	0.0	0.0
**S3**	69.2	7.5	**69.2**	**3.7**	61.5	1.5	46.2	0.7
**S4**	46.2	4.3	**30.8**	**2.9**	7.7	2.2	7.7	0.0
**S5**	76.9	7.7	61.5	5.1	**66.7**	**0.0**	30.8	0.0
**S6**	76.9	6.7	76.9	4.4	**61.5**	**2.2**	38.5	0.7
**S7**	38.5	8.1	**23.1**	**3.7**	7.7	3.0	0.0	3.0
**S8**	92.9	7.2	**92.9**	**3.6**	85.7	2.9	35.7	0.7
**S9**	**91.7**	**3.8**	83.3	1.5	66.7	0.8	25.0	0.0

**Table 4:** Results obtained in the pseudo-online analysis for different values of prior probability for class 1 (laser).

Subject	Probability π = 2			Probability π = 3			Probability π = 4		
	K	TP	FP/min	K	TP	FP/min	K	TP	FP/min
**S1**	5.0	53.8	3.7	5.0	46.2	2.2	4.0	69.2	2.9
**S2**	4.0	38.5	3.5	4.0	23.1	0.7	3.0	30.8	1.4
**S3**	5.0	69.2	3.0	4.0	69.2	3.0	3.0	69.2	3.7
**S4**	5.0	53.8	2.2	3.0	46.2	2.9	3.0	30.8	2.9
**S5**	4.0	66.7	2.6	4.0	66.7	0.0	4.0	66.7	0.0
**S6**	5.0	76.9	2.2	4.0	76.9	3.0	4.0	61.5	2.2
**S7**	4.0	15.4	3.7	5.0	15.4	3.0	3.0	23.1	3.7
**S8**	5.0	57.1	1.4	4.0	92.9	3.6	3.0	92.9	3.6
**S9**	4.0	83.3	2.3	3.0	91.7	2.3	2.0	91.7	3.8
**Mean ± σ**	4.6	57.2 ± 19.5	2.7 ± 0.8	**4.0**	**58.7 ± 26.3**	**2.3 ± 1.1**	3.0	58.7 ± 24.2	2.7 ± 1.2

**Table 5:** Results for capturing the time windows with the particularized maximum.

Subject	K = 2		K = 3		K = 4		K = 5	
	TP	FP/min	TP	FP/min	TP	FP/min	TP	FP/min
**S1**	76.9	11.7	69.2	6.6	**61.5**	**3.7**	38.5	2.9
**S2**	38.5	12.0	**23.1**	**2.8**	7.7	0.7	7.7	0.0
**S3**	69.2	11.9	61.5	6.7	53.8	4.5	**53.8**	**3.0**
**S4**	38.5	4.3	**23.1**	**2.9**	7.7	2.2	0.0	1.4
**S5**	83.3	8.5	66.7	6.8	**25.0**	**0.9**	16.7	0.0
**S6**	92.3	14.8	76.9	7.4	**61.5**	**3.0**	23.1	0.7
**S7**	8.3	4.4	**8.3**	**2.2**	8.3	1.5	0.0	0.7
**S8**	92.9	7.2	85.7	5.8	85.7	5.1	**71.4**	**2.9**
**S9**	91.7	3.8	**91.7**	**3.0**	58.3	1.5	33.3	0.0

**Table 6:** List of electrodes obtained with the automatic method for each subject. Electrodes coincidental with manual electrode selection in bold text.

Subject	Automatic Electrode Selection	Sample Instant (ms)
**S1**	**Fz**, **FC2**, **Cz**, CP1, Pz, P4, **P1**	482.5
**S2**	**Fz**, **FC2**, P3, **Pz**, **P4**	337.5
**S3**	**Fz**, FC1, **FCz**, **Cz**	386.7
**S4**	**Fz**, C4, P4, CP4, P1, P2, POz	710.0
**S5**	**Fz**, **FC2**, **P1**	698.0
**S6**	FC1, Cz, **P4**	789.2
**S7**	**Fz**, **FC1**, C4	148.3
**S8**	**Fz**, **FC1**, CP4, P1	151.7
**S9**	**Fz**, P3, P4, **P1**, POz	485.0

**Table 7:** Comparison between the results obtained with the automatic electrode selection and with the manual selection.

	Optimal Case with Automatic Selection			Optimal Case with Manual Selection		
Subject	K	TP	FP/min	K	TP	FP/min
**S1**	4	61.5	2.9	5	46.2	2.2
**S2**	3	46.2	3.5	4	23.1	0.7
**S3**	4	69.2	3.0	4	69.2	3.0
**S4**	5	30.8	2.9	3	46.2	2.9
**S5**	4	83.3	2.6	4	66.7	0.0
**S6**	4	61.5	0.0	4	76.9	3.0
**S7**	-	-	-	5	15.4	3.0
**S8**	4	100.0	3.6	4	92.9	3.6
**S9**	3	69.2	3.8	3	91.7	2.3

**Table 8:** Analysis of the instant time detection (in seconds): IMUs vs. Brain-Machine Interface (BMI), which indicates prediction time before physical stop of the user; and BMI vs. laser appearance, which indicates how much time the BMI needs in order to detect the stop after the obstacle appears suddenly through the laser.

Subject	IMUs Detection–BMI Detection	BMI Detection–Obstacle Appearance
**S1**	0.8 ± 0.1	1.2 ± 0.1
**S2**	1.9 ± 0.1	1.1 ± 0.2
**S3**	0.7 ± 0.2	1.2 ± 0.1
**S4**	0.7 ± 0.4	1.5 ± 0.4
**S5**	0.9 ± 0.5	1.3 ± 0.5
**S6**	0.8 ± 0.2	1.2 ± 0.2
**S7**	0.4 ± 0.0	0.9 ± 0.0
**S8**	0.8 ± 0.2	0.9 ± 0.1
**S9**	0.7 ± 0.2	1.2 ± 0.1
**Mean ± σ**	0.9 ± 0.2	1.2 ± 0.2

**Table 9:** Comparison between the bests results obtained with previous research.

Previous Research [17]				Current Research				
Subject	K	TP (%)	FP/min	Subject	M/A	K	TP (%)	FP/min
S1	4	28.6	8.0	S1	A	4	61.5	2.9
S2	4	42.9	2.1	S2	A	3	46.2	3.5
S3	2	42.9	8.5	S3	A	4	69.2	3.0
S4	2	7.1	2.7	S4	M	3	46.2	2.9
S5	2	28.6	11.9	S5	A	4	83.3	2.6
Mean ± σ		30.0 ± 14.6	6.7 ± 4.2	S6	A	4	61.5	0.0
				S7	M	5	15.4	3.0
				S8	A	4	100.0	3.6
				S9	M	3	91.7	2.3
				Mean ± σ			63.9 ± 26.2	2.6 ± 1.1
